# Whole Genome Sequence of the Non-Microcystin-Producing Microcystis aeruginosa Strain NIES-44

**DOI:** 10.1128/genomeA.00135-15

**Published:** 2015-03-19

**Authors:** Kunihiro Okano, Naoyuki Miyata, Yasuo Ozaki

**Affiliations:** Department of Biological Environment, Faculty of Bioresource Sciences, Akita Prefectural University, Akita City, Akita, Japan

## Abstract

Microcystis aeruginosa is a typical algal bloom-forming cyanobacterium. This report describes the whole-genome sequence of a non-microcystin-producing strain of Microcystis aeruginosa, NIES-44, which was isolated from a Japanese lake.

## GENOME ANNOUNCEMENT

Microcystis aeruginosa is a typical algal bloom-forming cyanobacteria, one of several cyanobacteria known to produce the potent hepatotoxin microcystin (1, 2). Harmful algal blooms (HABs) caused by these toxic cyanobacteria have become a problem in many developed countries. In Japan, adopting appropriate measures to combat these blooms is also an urgent issue. Recent studies have indicated a correlation between the seasonal prevalence of blue-green algae and genotypes of the genus Microcystis (3), and, as such, the genetic analysis of microcystin-producing and nonproducing strains is of prime importance. We report the whole-genome sequence of the non-microcystin-producing Microcystis aeruginosa strain NIES-44 (4).

Whole-genome sequencing was carried out using an Illumina HiSeq1000 system (Illumina, San Diego, CA, USA) with a paired-end library (400 bp) and a Roche/454 PE genome sequencer FLX (454 Life Sciences, Branford, CT, USA) with a mate-paired library (8 kb). HiSeq reads were assembled *de novo* using Velvet version 1.2.08 (https://www.ebi.ac.uk/~zerbino/velvet) and combined into a hybrid assembly with the 454 reads using GS *de novo* assembler version 2.8 (454 Life Sciences). Gaps between the resultant 375 contigs were closed using NESONI version 0.118 (http://www.vicbioinformatics.com/software.nesoni.shtml) and Platanus version 1.2.1 (http://platanus.bio.titech.ac.jp/platanus-assembler). The draft genome was annotated using the RAST server (http://rast.nmpdr.org/rast.cgi), which predicted protein-coding sequences (CDSs). Whole-genome homology mapping of various strains, including M. aeruginosa NIES-44, was performed using Gegenees version 2.2.1 (5).

The M. aeruginosa NIES-44 genome comprised 80 contigs (six scaffolds) and had a total length of 4,565,330 bp and a G+C content of 43.19%. It included 4,790 protein-coding sequences and 47 RNA-coding genes (i.e., two sets of rRNA genes and 41 tRNA genes). The annotation revealed that 2,614 CDSs exhibited homology to genes with known functions, and the remaining 2,176 genes were identified as encoding hypothetical proteins of unknown function.

The 16S rRNA sequences of M. aeruginosa NIES-44 were 99.73% homologous (1,489 bp) to those of M. aeruginosa strain NIES-843 (6). However, homology across the whole genome was only 70.04%, owing to deficits in microcystin synthetase (*mcy*) and nonribosomal peptide synthetase gene clusters. Since M. aeruginosa NIES-44 showed 64.69% homology to M. aeruginosa TAIHU98, in which *mcy* gene cluster deficits have been previously reported (7), these results support a high genetic diversity among non-microcystin-producing strains. Furthermore, only 37 of the numerous copies of the transposase gene identified in M. aeruginosa NIES-843 (6) and TAIHU98 (7) were found in NIES-44; it is therefore predicted that M. aeruginosa NIES-44 has a characteristic gene structure.

### Nucleotide sequence accession number.

This whole-genome shotgun project has been deposited at DDBJ/EMBL/GenBank under the accession number BBPA00000000 and refers to the first version that is described in this paper.
